# Primate embryoids: an in vitro model leading from a totipotency-like phase to early organogenesis

**DOI:** 10.1038/s41392-026-03003-w

**Published:** 2026-10-30

**Authors:** Aseem Tara, Wilfried A. Kues

**Affiliations:** Friedrich-Loeffler-Institut, Federal Research Institute for Animal Health, Biotechnology/Stem Cell Unit, Neustadt Rbge, Germany

**Keywords:** Totipotent stem cells, Regenerative medicine

In a recent seminal study published in *Cell*, Zheng et al.^1^ demonstrated a sophisticated embryoid model for primate embryogenesis to study development up to early organogenesis in vitro. This embryoid model is based on a totipotent-like cynomolgus blastomere-like stem cell (cTBLC) culture, which can be triggered to autonomously undergo pre- and post-implantation developmental features in a sequential manner reminiscent of these stages after fertilization.

**Fig. 1:**
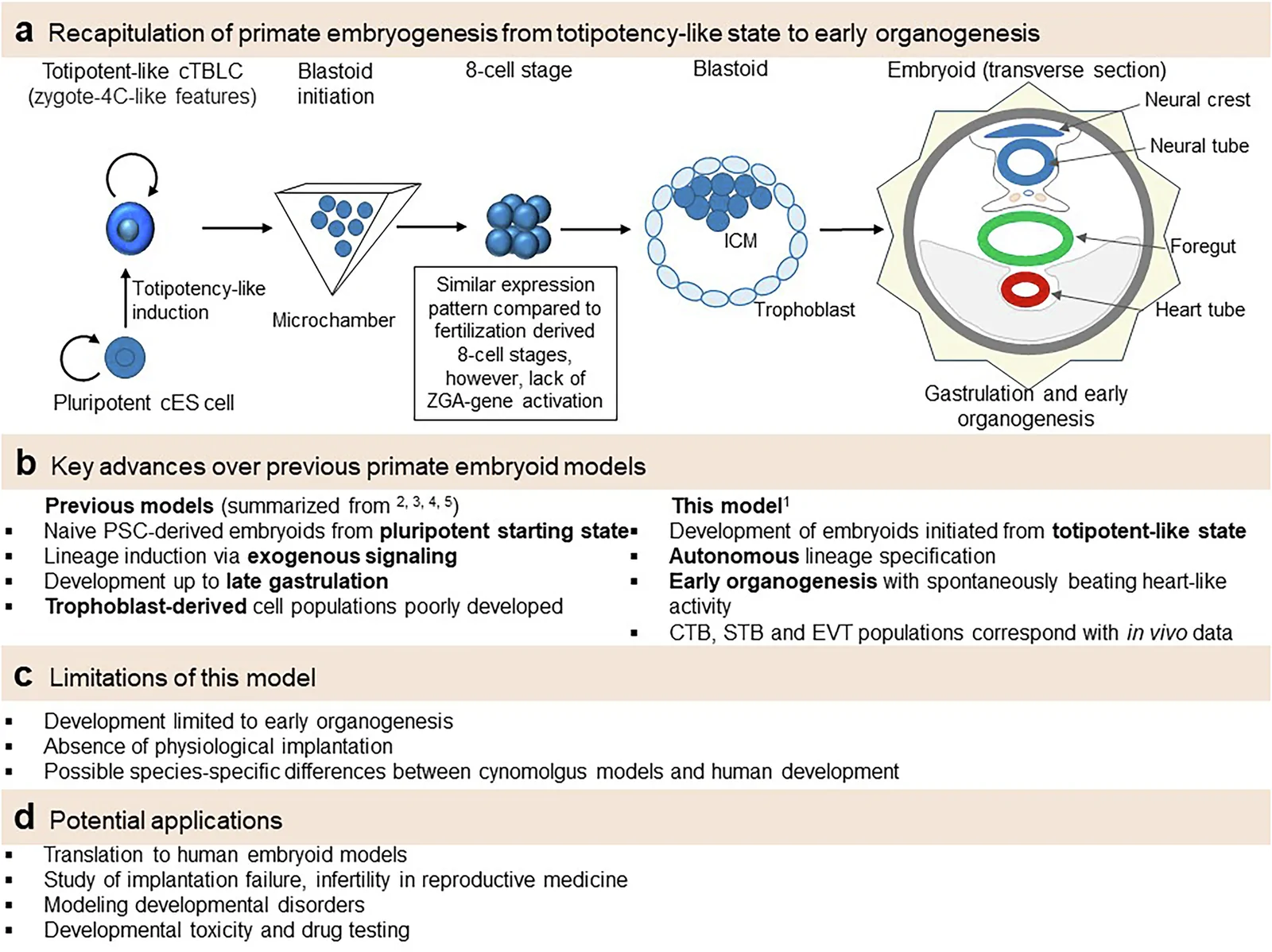
In vitro primate embryoid formation. **a** A totipotency-like developmental state can be induced in pluripotent cynomolgus ESCs by a cocktail of defined chemical compounds, including the spliceosome inhibitor pladienolide B. The resulting cTBLCs can be kept in the totipotent-like state under self-renewal conditions. Approximately 25 totipotent-like cTBLCs are seeded in a specific microchamber to trigger the formation of a blastoid-like structure, which is subsequently developed to early organogenesis reminiscent to these stages after fertilization and implantation. This approach allows the in vitro generation of post-implantation embryoids in 3D-culture systems. **b** A brief comparison with previous platforms is shown, and key advances of this study, such as the initiation of embryoid development from a totipotency-like state, are highlighted. **c** Limitations of the embryoid model, along with future potential applications, are summarized. **d** Perspective if this model can be translated to human cells and cells from other non-model species. CTB cytotrophoblast, STB syncytiotrophoblast, EVT extravillous trophoblast, ZGA zygotic genome activation

As current pluripotent stem cell (PSC)-based embryoid models recapitulate only partial aspects of early development, recreating a continuous developmental trajectory spanning blastomere to early primate embryogenesis remains challenging. Here, Zheng et al.^1^ generated primate embryoids from cTBLCs, which transitioned through an intermediate 8-cell/morula-like cell (8CMLC) state and progressed towards early organogenesis. Previous work has established human^2–4^, and primate^5^ embryoid systems capable of developing up to early gastrulation. The cTBLC-generated embryoids initiated ontogenesis from a totipotent-like state (Fig. 1), which distinguishes them from previous approaches employing lineage-restricted pluripotent cells.^2,5^ Building on their previous work establishing spliceosomal repression (see references in ref. ^1^) as a strategy to induce totipotency-associated blastomere-like states in mouse and human pluripotent stem cells (PSCs), they applied this approach in a non-human primate model to reprogram cynomolgus PSCs to generate cTBLCs. Current findings on these totipotent-like cells support an expanded developmental potential and a contribution to both embryonic and extra-embryonic cell lines; however, formal evidence of a bona fide totipotency with the complete development of an organism has not been shown. Here, they optimized a cocktail of different inhibitors and growth factors to improve the shift from a pluripotent to a totipotent-like cellular potential. The final LMYCIP conversion medium contained human leukemia inhibitory factor (LIF), minocycline (PARP1 inhibitor), Y-27632 (ROCK inhibitor), ChIR-99021 (GSK3 inhibitor), IWR-1 (Wnt inhibitor), and pladienolide B (splicing inhibitor).

cTBLCs exhibited reduced expression of pluripotency-associated markers, such as OCT4, SOX2, NANOG, and DNMT3B, compared to cPSCs. Transcriptomic analyses using bulk and single-cell RNA (scRNA-seq) sequencing further revealed a transition from a pluripotent state toward a totipotent blastomere-like state characterized by activation of zygote/2-4 cell-stage-specific genes. In blastocyst complementation assays, EGFP-labeled cTBLC cells were injected into murine 8-cell embryos; their descendants could be detected at low frequencies (1 per 10,000 murine cells) in embryonic and extra-embryonic tissues of these interspecies chimeras in post-implantation stages at day 10.5.

Unlike cPSCs, cTBLCs in culture displayed an inherent ability to generate organized blastoid structures in the absence of exogenous signaling cues, producing cavity-containing blastoids. A critical trigger for the switch from cTBLC proliferation in LMYCIP medium to preimplantation-like development was the seeding of ~25 cells in specific microwell chambers, which support 3-dimensional (3D) blastoid growth. In previous reports studying the differentiation of human^2–4^ and primate^5^ PSCs to blastoids, similar 3D-culture conditions were applied. The cTBLC-derived embryoids recapitulated key morphogenetic events associated with the 8-cell/morula stage, including compaction and polarization, as evidenced by E-cadherin redistribution and partitioning defective 6 homolog (PAR6) localization. Consistent with these morphological changes, scRNA-seq identified an 8CMLC population within cTBLC-derived structures, enriched for 8-cell/morula-stage gene signatures. Pseudotime analysis together with dynamic gene expression profiling further revealed a developmental trajectory from cTBLCs through 8CMLC stage toward epiblast (EPI), trophectodermal (TE), and primitive ectoderm (PrE)-like states, capturing key transcriptional transitions during preimplantation development. Although 8CMLCs exhibited transcriptional similarity to in vivo 8-cell/morula stages, they lacked activation of canonical zygotic genome activation (ZGA)-associated genes. The authors speculate that cTBLC-derived 8CMLCs may be decoupled from the regulatory requirements of ZGA/maternal to zygotic transition (MZT) due to their pre-established chromatin accessibility and transcriptional programs. Knocking down the ZGA regulator nuclear receptor subfamily 5 group A member 2 (NR5A2) did not disrupt 8CMLC formation or subsequent lineage specification, which seems to support an intrinsic developmental program independent of canonical ZGA regulation. However, alternative explanations, such as incomplete reprogramming or a residual transcriptional memory of the cTBLCs, can´t be excluded.

Under post-implantation culture conditions, cTBLC-derived embryoids progressed beyond the blastoid stage to form peri- and early post-implantation-like structures or gastruloids, including events like establishment of amnion- and yolk-sac-like compartments enclosed by a trophoblast-like layer, epiblast cell migration, and formation of the three germ layers. With extended culture under organogenesis-supporting conditions, these embryoids progressed beyond gastrulation to early organogenesis-like stages, exhibiting advanced developmental features including primordial germ cell (PGC) migration, regionalized gut formation, early neurulation, and cardiac development with beating heart-like activity. scRNA-seq analysis confirmed the emergence of diverse embryonic and extraembryonic lineages at the organogenesis stage, with cellular profiles closely resembling those of corresponding primate embryos derived from fertilization.

In summary, the data and methodology in the paper provided insights into cellular and molecular mechanisms in critical developmental stages of primate embryogenesis. Embryogenesis requires a well-orchestrated sequence of subsequent cell divisions and precisely tuned gene expression patterns. This work and previous studies on human and primate blastoid and gastrulation models^2–5^ surprisingly show that pluripotent and totipotent-like stem cells are sufficient for an ordered development of a blastocyst-like structure and beyond, pointing to an intrinsic capacity, which can be triggered under proper niche conditions. Limitations of this in vitro embryoid model are the absence of implantation and therefore the lack of maternal-placental interactions and a limited understanding of the long-term consequences, including a limited understanding of exactly how the physiological situation is recapitulated.

Should the translation of this roadmap to human cells prove feasible, it will have an impact on artificial reproduction techniques. The scarcity of human fertilized embryos for research, as well as strict legal and ethical restrictions have limited detailed investigations into why the majority of in vitro-produced blastocysts fail to implant. Human extended embryoid models will potentially allow, to test the effects of the genetic background of infertility patients or of known mutations, evaluating non-genetic factors, and to perform toxicity testing with regard to the peri-implantation phase. Incorporating maternal cells into the model could further advance the in vitro development.

Anyhow, the embryoid research direction will stimulate further investigations and raises a number of remaining questions and future challenges. Will it be possible to initiate blastoid formation from a single starter cell? Will it be possible to develop the current embryoid model into extended organogenesis? Will the provision of maternal-placental structures support the regular development in vitro? Will it be possible to develop blastoids, which are implantation competent and are able to initiate a full fetal development? And with regard to experiments with human embryoid structures, at which timepoint will they fall under legal restrictions? Freedom of research is an indispensable and valuable common good, but a scientific debate of the extent to which research with human embryoids is ethically justifiable should now be initiated.
